# Resilience as a protective factor against depression in informal caregivers

**DOI:** 10.3389/fpsyg.2024.1370863

**Published:** 2024-07-10

**Authors:** Fernando L. Vázquez, Vanessa Blanco, Elena Andrade, Patricia Otero, Ana M. Bueno, Miguel A. Simón, Ángela J. Torres

**Affiliations:** ^1^Department of Clinical Psychology and Psychobiology, University of Santiago de Compostela, Santiago de Compostela, Spain; ^2^Department of Evolutionary and Educational Psychology, University of Santiago de Compostela, Santiago de Compostela, Spain; ^3^Department of Social Psychology, Basic Psychology and Methodology, University of Santiago de Compostela, Santiago de Compostela, Spain; ^4^Department of Psychology, University of A Coruña, A Coruña, Spain; ^5^Department of Psychiatry, Radiology, Public Health, Nursing and Medicine, University of Santiago de Compostela, Santiago de Compostela, Spain

**Keywords:** resilience, depression, predictor, protective factor, informal caregivers, moderators

## Abstract

**Introduction:**

Although previous research has demonstrated that resilience can be protective against various mental health conditions such as depression, existing studies examining the relationship between resilience and depression have limitations. To our knowledge, the moderators of the relationship have not been examined. The aim of this study was to determine whether resilience acts as a protective factor against depression in informal caregivers and to examine potential moderators of the relationship between these variables.

**Methods:**

In this cross-sectional study, 554 randomly selected informal caregivers participated (86.8% women, average age = 55.3 years). Major depressive episode, depressive symptomatology, resilience, positive environmental reward, negative automatic thoughts, self-efficacy, and personality were assessed.

**Results:**

A total of 16.1% of informal caregivers met criteria for a depressive episode and 57.4% were at risk of developing depression. The average resilience score was 26.3 (*SD* = 7.6); 62.6% of participants were in the lower quartile of the resilience scale. The gender of the informal caregiver and self-efficacy acted as moderating variables in the relationship between resilience and depression. The impact of resilience on depressive symptoms was more pronounced in female informal caregivers, and increased as self-efficacy increased.

**Discussion:**

Based on these findings, programs aimed at preventing depression in informal caregivers should focus on promoting resilience, especially in women, and introduce strategies to enhance self-efficacy to increase their impact.

## 1 Introduction

The aging of the population is becoming the most dominant global demographic trend due to a decrease in birth rates, a notable increase in life expectancy, and the progression of large cohorts of people into advanced ages (Bloom and Zucker, 2023). According to the United Nations (United Nations, Department of Economic and Social Affairs, Population Division, 2022), the percentage of the worldwide population over 65 is expected to increase from 10% in 2022 to 16% by 2050. This growth will be especially marked in Europe and North America, where it is expected to rise from the current 18.7% to 26.9% by 2050.

Population aging is considered the greatest demographic challenge the world must face (Bloom and Zucker, 2023). Among the most important consequences are the implications for the sustainability of welfare systems, both health care and long-term care. Implications on health care include the increase in disability rates caused by various health conditions (Leist, 2018) and implications on long-term care include the services needed for people with reduced functionality, whether physical or cognitive, who require assistance for daily activities over a prolonged period (European Commission, 2015). According to the Organization for Economic Cooperation and Development (OECD, 2021), in 2019, of the countries that constitute the OECD, around 10.7% of people over 65 received long-term care.

Many people who need this type of care would prefer to stay in their homes as long as possible. In fact, 68% of those receiving long-term care in OECD countries did so in their own homes (OECD, 2021). These high figures highlight the important role of informal caregivers, who are typically family, close relatives, friends, or neighbors, who provide non-professional, unpaid care performing a wide variety of tasks, including emotional support and assistance (Triantafillou et al., 2010). 34.3% of the European population assumes this role, and 7.6% are intensive informal caregivers (those who care for 11 h a week or more) (Verbakel et al., 2017); and these figures are expected to increase due to the aforementioned population projections, which will result in an increased need for long-term care (Belmonte et al., 2023), mostly provided by informal caregivers (OECD, 2021).

Assuming the role of informal caregiver is not without consequences, especially in relation to mental health, with informal caregivers more likely to experience symptoms of anxiety, depression, burden, and lower life satisfaction (Revenson et al., 2016). A study analyzing cross-sectional data from the World Health Survey collected from 258,793 adults (Koyanagi et al., 2018), found that being an informal caregiver was associated with a higher likelihood of depression, sleep problems, and perceived stress.

In a recent systematic review (Janson et al., 2022), higher incidences (i.e., appearance of new cases) of severe stress, adjustment disorders, and depression in informal caregivers, compared to individuals who do not perform informal caregiving tasks, were found. Additionally, informal caregivers were more likely to have higher rates of depression, with prevalence rates ranging between 25.1% (Pan and Lin, 2022) and 42.3% (Loh et al., 2017; Geng et al., 2018; Collins and Kishita, 2020; Bedaso et al., 2022; Pan and Lin, 2022). However, these studies assessed the presence of depression using various evaluation instruments, often self-report tools, and did not provide information about the diagnoses of specific depressive symptoms and disorders.

Depression can have multidimensional effects on informal caregivers. There are effects on physical and mental health, including an increased risk of chronic diseases (Schulz and Eden, 2016) and suicide (Solimando et al., 2022) and reductions in quality of life (Montgomery et al., 2018). Depression can also lead to detrimental effects on the quality of the care they provide (Williamson et al., 2001), increasing the probability of abandoning the informal caregiver role and institutionalization of their loved one (Schoenmakers et al., 2010). Depression of the informal caregiver of individuals with dementia is also associated with a faster cognitive decline of the care recipient (Norton et al., 2013). Further, informal caregivers with depression often isolate themselves, withdrawing from their social and family networks. This isolation can reduce their available social support (Wang et al., 2016) and in turn, create a vicious circle that intensifies depressive symptoms. Finally, for informal caregivers who perform a paid work activity in addition to their caregiving, depression can lead to difficulties in work-life balance, loss of work productivity, and increased absenteeism (Fujihara et al., 2019; Beauchamp et al., 2023).

While the negative consequences of caregiving on the mental health of the informal caregiver have been extensively documented, not all informal caregivers are negatively affected by the caregiving situation. Various studies have analyzed the effect of protective factors against depression in this population, including variables such as social support (e.g., Greenwell et al., 2015; Gutiérrez-Sánchez et al., 2023), personality (e.g., Greenwell et al., 2015), self-esteem, optimism, and perceived control (e.g., Chung et al., 2016). Other variable that could play a central role in maintaining adequate levels of wellbeing and functioning in informal caregivers is resilience (Fernández-Lansac and Crespo, 2011). Resilience can be defined as the capacity to positively adapt in situations of stress, trauma, or adversity, without developing limitations in physical, psychological, or social domains (Luthar et al., 2000; Tempski et al., 2015); it represents a paradigm shift in psychology with a greater focus on wellbeing and protective factors rather than solely on mental illnesses and risk factors (Connor and Davidson, 2003). The conceptualization of this construct has been inconsistent, and there is still debate about whether it represents a trait, a set of personal characteristics, or a system (Fernández-Lansac and Crespo, 2011; Mukherjee and Kumar, 2017). In the context of the current study, resilience is defined as a set of individual characteristics. From this perspective, there are a series of qualities associated with it, including, among others, self-confidence, discipline, perseverance, flexibility, emotional resistance, problem-solving, optimism, empathy, self-esteem, goal orientation, and low anxiety (Kumpfer and Hopkins, 1993; Giordano, 1997; Martin and Marsh, 2006). All these modifiable characteristics allow individuals to face stressful or adverse situations in a more positive way.

In recent years, the study of resilience in the population of informal caregivers has aroused increasing interest, resulting in a number of studies that have been included in several review articles and meta-analyses (e.g., Iacob et al., 2020; Palacio et al., 2020; McKenna et al., 2022). Specifically, various studies have analyzed the relationship between resilience and depression in informal caregivers of people with various health conditions, especially dementia (e.g., O'Rourke et al., 2010; Fernández-Lansac et al., 2012; Bitsika et al., 2013; Dias et al., 2016, 2020; Pastor-Cerezuela et al., 2016; Sutter et al., 2016; Timmons et al., 2016; Halstead et al., 2018; Hwang et al., 2018; Pessotti et al., 2018; Jones et al., 2019; Kimura et al., 2019; Bermejo-Toro et al., 2020; Tyler et al., 2020; Vatter et al., 2020). Most of them found a significant inverse correlation between resilience and depressive symptoms (Fernández-Lansac et al., 2012; Timmons et al., 2016; Pessotti et al., 2018; Kimura et al., 2019; Dias et al., 2020; Tyler et al., 2020; Vatter et al., 2020). Other studies found that resilience acted as a protective or compensatory factor, directly reducing negative outcomes, so that higher levels of resilience predicted lower levels of depression (Dias et al., 2016; Pastor-Cerezuela et al., 2016; Sutter et al., 2016; Halstead et al., 2018; Bermejo-Toro et al., 2020). Three studies (Bitsika et al., 2013; Hwang et al., 2018; Jones et al., 2019) found that informal caregivers with lower levels of resilience had higher levels of depression, or vice versa. One study (O'Rourke et al., 2010) found that resilience predicted changes in depressive symptoms 1 year later.

However, none of these previous studies reported on moderating variables between resilience and depression. Moderators are intervening variables that affect the direction and/or strength of the relationship between an independent variable (in this case, resilience) and a dependent variable (depression), reducing, increasing, nullifying, or reversing it (MacKinnon, 2011). Moderation analyses can provide greater detail about the relationship between variables. For example, in this study, resilience can be divided into subgroups that establish domains of optimized effects related to depression, indicating when, how, and for whom the effect of resilience (i.e., the independent variable) on depression (i.e., the dependent variable) will be greatest or least. Although research on resilience and depressive symptoms in other populations is also limited, the findings highlight the need to study potential moderators between these two variables (Lau, 2022). Existing data suggest that some sociodemographic variables, such as gender (Wen et al., 2023), and clinical variables, such as the presence of ruminative thoughts (Xu et al., 2021), could modify the relationship between resilience and depression.

Furthermore, existing research on the relationship between resilience and depression in informal caregivers is still insufficient and suffers from many limitations arising from conceptual confusion and methodological problems. Among them, very few studies have started with a clear definition of resilience (O'Rourke et al., 2010; Halstead et al., 2018; Dias et al., 2020); most of them have small sample sizes (Fernández-Lansac et al., 2012; Dias et al., 2016, 2020; Timmons et al., 2016; Pessotti et al., 2018; Kimura et al., 2019; Bermejo-Toro et al., 2020), utilizing convenience samples (Bitsika et al., 2013; Dias et al., 2016, 2020; Timmons et al., 2016; Jones et al., 2019; Kimura et al., 2019), samples of informal caregivers of people with Alzheimer's or other dementias and no other physical or mental disability (Fernández-Lansac et al., 2012; Dias et al., 2016, 2020; Pessotti et al., 2018; Jones et al., 2019; Kimura et al., 2019), and a lack of standardized diagnostic criteria such as DSM-5-TR or ICD-11 for the diagnosis of depression (Jones et al., 2019; Bermejo-Toro et al., 2020; Dias et al., 2020; Vatter et al., 2020). These issues limit the generalizability of the findings.

The main objective of the current study was to analyze the level of resilience and its role as a protective factor against depression in a randomly selected sample of informal caregivers, as well as sociodemographic information and information about their caregiving situation, and clinical variables that may act as moderators of the relationship between resilience and depression.

## 2 Materials and methods

### 2.1 Design and participants

A cross-sectional design was used. The study was conducted between September 19 and October 31, 2023. The sample was drawn through random sampling from the informal caregivers registered in public institutions and associations for people with chronic diseases in Galicia (Spain), through which the recruitment of the sample was conducted.

The inclusion criteria for participants were: (a) 18 years of age or older; (b) being the main informal caregiver of a person in a situation of dependence recognized by the competent public administration; and (c) having been providing care for at least 6 months. The exclusion criteria were: (a) not residing permanently with the person being assisted; (b) suffering from a condition that could hinder the performance of the assessment tasks; or (c) participating in any psychological intervention or receiving psychopharmacological treatment.

A random sample of 576 informal caregivers was selected. The sample size was calculated based on an estimated prevalence of depression of 6% (estimation based on scientific literature and a previous pilot study), an alpha risk of 0.05, precision ± 2%, alpha error = 5%, and an expected 6% sample loss proportion. The response rate was 99.1%. A total of 5 informal caregivers declined to participate in the study, and 17 did not meet the eligibility criteria (1 had not provided care for the minimum time required, 2 were not residing permanently with the care recipient, and 14 were participating in a psychological intervention or receiving psychopharmacological treatment), resulting in a final sample of 554 participants (86.8% women) with an average age of 55.3 years (*SD* = 11.8).

The study was conducted in accordance with the principles of the Declaration of Helsinki and ensured compliance with Organic Law 3/2018, of December 5, on Personal Data Protection and guarantee of digital rights (Organic Law, 2018). Additionally, approval was obtained from the Bioethics Committee of the University of Santiago de Compostela (Cod. USC 49/2023). Participation was completely voluntary, with no economic or other incentives, and all participants gave their written informed consent.

### 2.2 Variables and assessment instruments

The variables included in the study were classified into three categories: predictors (resilience), outcomes (diagnosis of major depressive episode and depressive symptoms [which was the outcome variable in the moderation analyses]), and moderating variables (variables related to caregiving, positive environmental reward, negative automatic thoughts, self-efficacy, and personality). The following instruments were used to evaluate them.

#### 2.2.1 Variables related to caregiving

An ad hoc questionnaire was used to collect information on the following variables: (a) sociodemographic variables of the caregiver (gender, age, marital status, education level, main activity, and family monthly income); (b) sociodemographic variables of the person in the situation of dependence (gender, age); and (c) variables of the caregiving situation (relationship between the caregiver and the cared-for person, diagnosis of the care recipient, years of duration of the caregiving situation, and daily hours dedicated to care). Interrater reliability between the estimates made by the evaluators and the supervisors was exact (*K* = 1).

#### 2.2.2 Diagnosis of depressive episode

The Structured Clinical Interview for DSM-5-Clinician Version (SCID-5-CV, First et al., 2015) was used to diagnose major depressive episodes. This interview is the most common diagnostic tool from clinical practice for diagnosing depressive episodes according to the DSM-5 and must be administered by a clinician. For this study, the presence of a major depressive episode (MDE), corresponding to module A of the SCID-5-CV, was used. Questions were based on the respective DSM-5 criteria (e.g., loss of interest, thoughts about death), which are to be rated as present or absent. The SCID severity scales for the current major depressive episode showed high internal consistency (Cronbach's alpha = 0.91), test-retest reliability, and concurrent and predictive validity (Shankman et al., 2018). Interrater reliability between the estimates made by the evaluators and the supervisors (*K*) was 0.95.

#### 2.2.3 Depressive symptomatology

Depressive symptomatology was assessed using the Center for Epidemiologic Studies Depression Scale (CES-D; Radloff, 1977; Spanish version by Vázquez et al., 2007). It is a self-reported 20-item instrument providing a continuous score reflecting the level of depressive symptomatology over the past week. Each of the 20 items (e.g., I felt sad, I felt lonely) is rated on a four-option Likert scale ranging from 0 (rarely or none of the time) to 3 (most of the time), with a range from 0 to 60 (a higher score corresponds to greater depressive symptomatology). A score of 16 or higher is considered indicative of the risk of clinical depression (Lewinsohn et al., 1997). The internal consistency of the Spanish version (Cronbach's alpha) was 0.89. A score of 26 was a suitable cut-off for screening purposes, providing a sensitivity of 0.906 and a specificity of 0.918.

#### 2.2.4 Resilience

Resilience was assessed using the 10-item Connor-Davidson Resilience Scale (CD-RISC-10; Campbell-Sills and Stein, 2007; Spanish version by Blanco et al., 2019a). It is a self-reported 10-item (e.g., I am able to adapt to changes, I see myself as a strong person) instrument that assesses an individual's ability to cope with adversity. Each item is rated on a five-point Likert scale ranging from 0 (not true at all) to 4 (true most of the time). The score range is from 10 to 40, with higher scores indicating higher levels of resilience. According to Campbell-Sills et al. (2009), using a general American adult population (*N* = 764), percentiles were the following for the CD-RISC-10 (median score = 32): (1) lowest 25%, corresponding to the first quartile (Q1), scores ranged from 0–29; (2) 25–50th percentiles, corresponding to the second quartile (Q2), scores ranged from 30–32; (3) 50–75th percentiles, corresponding to the third quartile (Q3) scores ranged from 33–36; and (4) highest 25%, corresponding to the fourth quartile, scores ranged from 37–40. The internal consistency of the Spanish version was 0.86. A score of 23 was a suitable cut-off point for discriminating caregivers with depression (sensitivity = 70.0%, specificity = 68.2%). Its convergent validity with other measures of self-esteem, social support and emotional distress ranged from 0.23 to 0.42.

#### 2.2.5 Positive environmental reward

Positive environmental reward was assessed with the Environmental Reward Observation Scale (EROS; Armento and Hopko, 2007; Spanish version by Barraca and Pérez-Álvarez, 2010). It is a self-reported 10-item (e.g., Many activities in my life are enjoyable, Other people seem to have more fulfilling lives) tool that measures the degree of reward provided by the environment. Participants must respond to each item on a four-point Likert scale, from 1 (strongly disagree) to 4 (strongly agree), based on how applicable each item is to them. The range is from 10 to 40, and higher scores indicate a greater degree of contingent positive environmental reward. The internal consistency of the Spanish version was 0.86. The convergent validity with other instruments assessing depressive symptoms, negative automatic thoughts, behavioral activation, anxiety, and experiential avoidance ranged from 0.48 to −0.80.

#### 2.2.6 Negative automatic thoughts

For the assessment of participants' negative thoughts, the Automatic Thoughts Questionnaire-Negative (ATQ-N; Hollon and Kendall, 1980; Spanish version by Otero et al., 2017) was used. This is a self-reported 30-item (e.g., I have disappointed people, Nothing will ever be good) questionnaire that assesses negative thoughts associated with depression over the past week. The individual must indicate the frequency with which a series of thoughts have suddenly arisen in their head during the last week, on a five-point scale, from 1 (never) to 5 (always). The total score can range from 30 to 150, and higher scores indicate a greater frequency of negative automatic thoughts. The internal consistency for the Spanish version was 0.96. A score of 52 proved to be an appropriate cut-off point to distinguish depressed caregivers from non-depressed informal caregivers (sensitivity = 80.9%; specificity = 75.5%). The convergent validity with another instrument assessing depressive symptoms was 0.68.

#### 2.2.7 Self-efficacy

Self-efficacy was assessed using the General Self-Efficacy Scale (GSES; Schwarzer and Jerusalem, 1995; Spanish version by Blanco et al., 2019b). This self-reported scale consists of 10 items (e.g., I can solve difficult problems if I try hard enough; If I find myself in a difficult situation, I usually come up with what I should do) that evaluate self-efficacy on a Likert scale from 1 (incorrect) to 4 (correct). The score range varies from 10 to 40, with a higher score indicating a greater sense of self-efficacy. The internal consistency of the original Spanish version was 0.90. A score of ≤ 28 proved to be an appropriate cut-off point to distinguish informal caregivers with or without depression (sensitivity = 71.0%; specificity = 53.2%). The convergent validity with measures of neuroticism and extraversion was −0.35 and 0.36, respectively. The criterion validity with measures of depressive symptoms and negative automatic thoughts was −0.32 and −0.27, respectively.

#### 2.2.8 Personality

Personality was assessed using the Eysenck Personality Questionnaire Revised-Abbreviated (EPQR-A; Francis et al., 1992), a self-administered questionnaire consisting of 24 items and 4 subscales of 6 items each with two response options (yes or no). Three subscales measure personality traits (Neuroticism [some examples of items from this subscale are Do you frequently have mood swings?, or Do you feel fed-up?], Extraversion [some examples of items from this subscale are Do you frequently have mood swings?, or Do you feel fed-up?], Psychoticism [some examples of items from this subscale are Would you take drugs that could have unknown or dangerous effects?, or Do you believe it is better to follow society's rules than your own?]), and a fourth measures the tendency to respond in a socially desirable manner (Sincerity). Scores on each subscale range from 0 to 6, with higher scores indicating a greater presence of the trait. In various samples of the study, Francis et al. (1992) found satisfactory internal consistency indices for the three subscales (α = 0.70–0.77, α = 0.74–0.84 and α = 0.59–0.65, respectively). Concurrent validity was assessed by analyzing the correlations of the subscales with the parent forms of the original EPQR scales. Correlations ranged from 0.93 to 0.95 for the extraversion subscale, 0.92 to 0.94 for neuroticism, 0.80 to 0.87 for psychoticism, and 0.90 to 0.92 for sincerity.

### 2.3 Procedure

A detailed protocol was developed outlining the study's objectives, design and framework, participants (target population, accessible population, inclusion/exclusion criteria, and recruitment), measures, biases (non-response, recall, and selection), data analysis strategy, quality control, data management, scheduling, and ethical aspects.

Five psychologists were trained to perform the clinical assessments. They received 20 h of training, including theoretical and practical seminars covering evaluation of the informal caregiver population, interview and questionnaire administration, and role-playing.

A pilot study with 55 randomly selected informal caregivers from the institutions where participants would later be recruited was conducted to assess feasibility, interviewer competence, and to help estimate the sample size of the current study. All evaluations were recorded for performance assessment and feedback. No modifications were required. A prevalence of MDE of 14.5% was found.

Subsequently, selected informal caregivers were contacted by mail, email, or phone, informed about the study, and invited to participate. Strategies were followed to minimize participant loss as recommended by Newman et al. (2023), which included, among others: sending reminders of the assessment date, not using invasive information collection procedures, and presenting the study to informal caregivers in an engaging manner. Participants were evaluated by phone.

First, sociodemographic information and information about caregiving situation were collected, then the MDE diagnostic criteria were evaluated with SCID-5-VC. Self-reported resilience, positive environmental reward, negative thoughts, self-efficacy, personality, and depressive symptomatology was then collected. The evaluation lasted approximately 40 min. Evaluators were supervised weekly by a training expert, who also analyzed a random sample (10%) of evaluations.

### 2.4 Data analysis

All statistical analyses were conducted using SPSS for Windows (version 28.0) and R (R Core Team, 2023). The choice of statistical tests to determine statistical significance considered: (a) the purpose of the analysis, (b) the characteristics of the variables, (c) the application conditions of each test, (d) the study design, and (e) the number of groups. Frequency distributions and descriptive statistics (means, standard deviations and range) were used to describe the potential moderators, (variables related to caregiving and clinical characteristics of the sample [positive environmental reward, negative automatic thoughts, self-efficacy, and personality]); the outcomes (prevalence of depression and depressive symptomatology); and the predictors (resilience).

Independent samples *t*-tests, Pearson correlations, and one-way analysis of variances (ANOVAs) were used to analyze the relationship between the potential moderators (variables related to caregiving and clinical characteristics of the sample [positive environmental reward, negative automatic thoughts, self-efficacy, and personality]) with resilience. Bonferroni *post hoc* contrasts were used in ANOVAs when differences between groups were found.

To examine the relationship between resilience and depression, multiple linear regression analyses were conducted, following the strategies proposed by Domenéch and Navarro (2006). The impact of potential moderators influencing the relationship between resilience and depressive symptomatology was determined using linear regression analysis. The Baron and Kenny (1986) model, O = α + β1T + β2M + β3TM, was used to assess the effect of the potential moderator, where O represents depressive symptomatology, T resilience, M the potential moderator, and TM the interaction between resilience and the potential moderator. Potential moderators included variables related to caregiving and clinical characteristics of the sample (positive environmental reward, negative automatic thoughts, self-efficacy, and personality).

## 3 Results

### 3.1 Variables related to caregiving

The main sociodemographic characteristics of the informal caregiver, the person in a situation of dependence, and the caregiving situation of the final sample (*n* = 554) are presented in Table 1. Of the total sample, 86.8% (*n* = 481) were women, with an average age of 55.3 years (*SD* = 11.8; range 25–86), 71.8% (*n* = 398) had a partner; 54.5% (*n* = 302) had primary education, 18.8% (*n* = 104) secondary education, 13.2% (*n* = 73) university education, 13.2% (*n* = 73) no formal education but could read and write, and 0.4% (*n* = 2) were illiterate; 80.1% (*n* = 317) were not self-employed or employed by others; 58.1% (*n* = 322) had monthly family incomes between 1,000 and 1,999 €, 24.7% (*n* = 137) had < 1,000 €, and 17.1% (*n* = 95) had 2,000 € or more. Regarding the person in a situation of dependence, 62.8% (*n* = 348) were women, with an average age of 63.1 years (*SD* = 31.2; range 2–101). For 30.1% (*n* = 167) of the informal caregivers, the diagnosis of the care recipient was dementia; 37.7% (*n* = 209) cared for their father or mother for an average of 12.9 years (*SD* = 8.8), and 15.8 (*SD* = 3.9) hours per day.

**Table 1:** Variables related to caregiving (*N* = 554).

**Variables**	***N***	**%**
**Informal caregiver variables**		
**Gender**		
Male	73	13.2
Female	481	86.8
**Age (years)**		
*M (SD)*	55.3 (11.8)	
Range	25–86	
**Marital status**		
Without a partner	156	28.2
With a partner	398	71.8
**Education level**		
Illiterate	2	0.4
No formal education, but can read and write	73	13.2
Primary education	302	54.5
Secondary education	104	18.8
University education	73	13.2
**Main activity**		
Self-employed or employed by others	110	19.9
Others	444	80.1
**Family monthly income**		
< 1,000 €	137	24.7
1,000–1,999 €	322	58.1
≥2,000 €	95	17.1
**Dependent person variables**		
Gender		
Male	206	37.2
Female	348	62.8
**Age (years)**		
*M (SD)*	63.1 (31.2)	
Range	2–101	
**Caregiving situation variables**		
**Relationship to the care recipient**		
Parent	209	37.7
Other	345	62.3
**Diagnosis of the care recipient**		
Dementias	167	30.1
Other	387	69.9
**Duration of care (years)**		
*M (SD)*	12.9 (8.8)	
Range	0.9–51	
**Daily hours dedicated to care**		
*M (SD)*	15.8 (3.9)	
Range	3–24	

### 3.2 Clinical characteristics of the sample

The main clinical characteristics of the final sample (*n* = 554) are presented in Table 2. The average scores were 28.0 (*SD* = 5.3) for positive environmental reward, 50.0 (*SD* = 21.2) for negative thoughts, 29.5 (*SD* = 6.3) for self-efficacy, 3.0 (*SD* = 2.0) for neuroticism, 3.7 (*SD* = 1.9) for extraversion, and 1.3 (*SD* = 1.0) for psychoticism.

**Table 2:** Clinical characteristics for the sample (*n* = 554).

**Variables**	
**Positive environmental reward**	
*M (SD)*	28.0 (5.3)
Range	15–40
**Negative thoughts**	
*M (SD)*	50.0 (21.2)
Range	30–132
**Self-Efficacy**	
*M (SD)*	29.5 (6.3)
Range	Oct-40
**Neuroticism**	
*M (SD)*	3.0 (2.0)
Range	0–6
**Extraversion**	
*M (SD)*	3.7 (1.9)
Range	0–6
**Psychoticism**	
*M (SD)*	1.3 (1.0)
Range	0–4

### 3.3 Prevalence of depression and depressive symptomatology

According to the SCID-CV results, 16.1% (*n* = 89) of participants were experiencing a major depressive episode. The most frequently reported symptoms by caregivers with a major depressive episode were (see Table 3): depressed mood (92.1%); insomnia (87.6%); fatigue or loss of energy (84.3%); and thoughts about death (84.3%).

**Table 3:** Most frequent symptoms of informal caregivers with depressive episodes (*n* = 89).

**Item**	**Yes**		**No**	
	***n***	***%***	***n***	***%***
1. Depressed mood	82	92.1	7	7.9
2. Markedly diminished interest or pleasure	71	79.8	18	20.2
3a. Decreased/increased appetite	47	52.8	42	47.2
3b. Weight loss	19	21.3	70	78.7
3c. Weight gain	52	58.4	37	41.6
4a. Insomnia	78	87.6	11	12.4
4b. Hypersomnia	12	13.5	77	86.5
5a. Psychomotor retardation	41	46.1	48	53.9
5b. Psychomotor agitation	40	44.9	49	55.1
6. Fatigue or loss of energy	75	84.3	14	15.7
7. Feelings of worthlessness or excessive or inappropriate guilt	28	31.5	61	68.5
8a. Difficulty concentrating or making decisions	68	76.4	21	23.6
8b. Slow or confused thoughts	57	64.0	32	36.0
9a. Thoughts about death	75	84.3	14	15.7
9b. Desire to die	29	32.6	60	67.4
9c. Suicide thoughts	16	18.0	73	82.0
9d. Suicide attempt	3	3.4	86	96.6

The average score for depressive symptomatology (16 is indicative of risk) was 18.3 (*SD* = 11.4), ranging from 0 to 53, meaning that 57.4% (*n* = 318) of informal caregivers in the sample were at risk of depression.

### 3.4 Resilience

#### 3.4.1 Resilience levels

The average resilience score was 26.3 (*SD* = 7.6). Using the cut-off points of 0–29, 30–32, 33–36, and 37–40 to identify quartiles, it was found that 62.6% (*n* = 347) were in Q1, 15.0% (*n* = 83) in Q2, 13.0% (*n* = 72) in Q3, and 9.4% (*n* = 52) in Q4. The frequency distribution of the informal caregivers' responses to the CD-RISC-10 Scale items is shown in Table 4. The items for which caregivers most frequently reported that 'It is true most of the time' were: *I try to recover after an illness or difficulty* (59.7%), *I am able to adapt to changes* (50.9%), and *I see myself as a strong person* (41.2%).

**Table 4:** Responses of the informal caregivers to the CD-RISC-10 (*n* = 554).

**Item**	**Not true at all**		**Rarely true**		**Sometimes true**		**Often true**		**True most of the time**	
	***n***	***%***	***n***	***%***	***n***	***%***	***n***	***%***	***n***	***%***
I am able to adapt to changes	9	1.6	15	2.7	113	20.4	135	24.4	282	50.9
I can handle anything that comes my way	23	4.2	43	7.8	204	36.7	176	31.8	108	19.5
I try to see the humorous side of problems	59	10.6	84	15.2	166	30.0	119	21.5	126	22.7
Coping with stress can make me stronger	51	9.2	62	11.2	135	24.4	158	28.5	148	26.7
I try to recover after an illness or difficulty	5	0.9	13	2.4	53	9.6	152	27.4	331	59.7
I can achieve my goals despite obstacles	12	2.2	45	8.1	191	34.5	174	31.4	132	23.8
I can stay focused under pressure	99	17.9	106	19.1	175	31.6	97	17.5	77	13.9
I don't easily get discouraged by failures	51	9.2	71	12.8	161	29.0	120	21.7	151	27.3
I see myself as a strong person	41	7.4	42	7.6	122	22.0	121	21.8	228	41.2
I can handle unpleasant emotions	46	8.3	59	10.6	175	31.6	126	24.5	138	24.9

#### 3.4.2 Variables associated with the informal caregiver's resilience

Average resilience scores based on informal caregiver, care recipient, caregiving situation, and clinical variables, along with statistical analyses to explore group differences and associated *p*-values, are detailed in Table 5. Significant differences in resilience were observed based on informal caregiver's gender [*t*_(552)_ = 3.084, *p* = 0.002], and a significant direct correlation was found between informal caregiver's age and resilience (*r* = 0.098, *p* = 0.021). A significant direct correlation between time spent caregiving and resilience (*r* = 0.094, *p* = 0.027) was also noted. Clinically, significant direct correlations of resilience with positive environmental reward (*r* = 0.499, *p* < 0.001), self-efficacy (*r* = 0.664, *p* < 0.001), and extraversion (*r* = 0.359, *p* < 0.001), and significant inverse correlations with negative thoughts (*r* = −0.380, *p* < 0.001) and neuroticism (*r* = −0.508, *p* < 0.001) were found. No relationship was found between other informal caregiver, care recipient, caregiving situation, or clinical variables and resilience.

**Table 5:** Variables associated with resilience (*n* = 554).

**Variables**	***M (SD)***	***F/t/r***	***p***
**Variables related to caregiving**			
**Informal caregiver variables**			
**Gender**			
Male	28.9 (7.1)	3.084	0.002
Female	25.9 (7.7)		
Age (years)		0.098	0.021
**Marital status**			
Without a partner	26.1 (7.9)	−0.34	0.734
With a partner	26.4 (7.6)		
**Education level**			
Illiterate	22.5 (3.5)	1.411	0.229
No formal education, but can read and write	26.9 (8.4)		
Primary education	26.1 (7.6)		
Secondary education	25.5 (7.6)		
University education	27.9 (6.8)		
**Main activity**			
Self-employed or employed by others	26.3 (7.7)	0.005	0.996
Others	26.3 (7.6)		
**Family monthly income**			
< 1,000 €	26.0 (7.8)	1.267	0.283
1,000–1,999 €	26.1 (7.8)		
≥2,000 €	27.4 (6.9)		
**Dependent person variables**			
**Gender**			
Male	26.3 (7.2)	0.063	0.95
Female	26.3 (7.9)		
Age (years)		−0.032	0.45
**Caregiving situation variables**			
**Relationship to the care recipient**			
Parent	25.5 (7.7)	−1.849	0.065
Other	26.8 (7.6)		
**Diagnosis of the care recipient**			
Dementias	26.7 (7.9)	0.815	0.415
Other	26.1 (7.6)		
Duration of care (years)		0.094	0.027
Daily hours dedicated to care		−0.063	0.139
**Clinical variables**			
Positive environmental reward		0.499	< 0.001
Negative thoughts		−0.38	< 0.001
Self-efficacy		0.664	< 0.001
Neuroticism		−0.508	< 0.001
Extraversion		0.359	< 0.001
Psychoticism		−0.026	0.54

### 3.5 Resilience as a protective factor against depression

**Figure 1:**
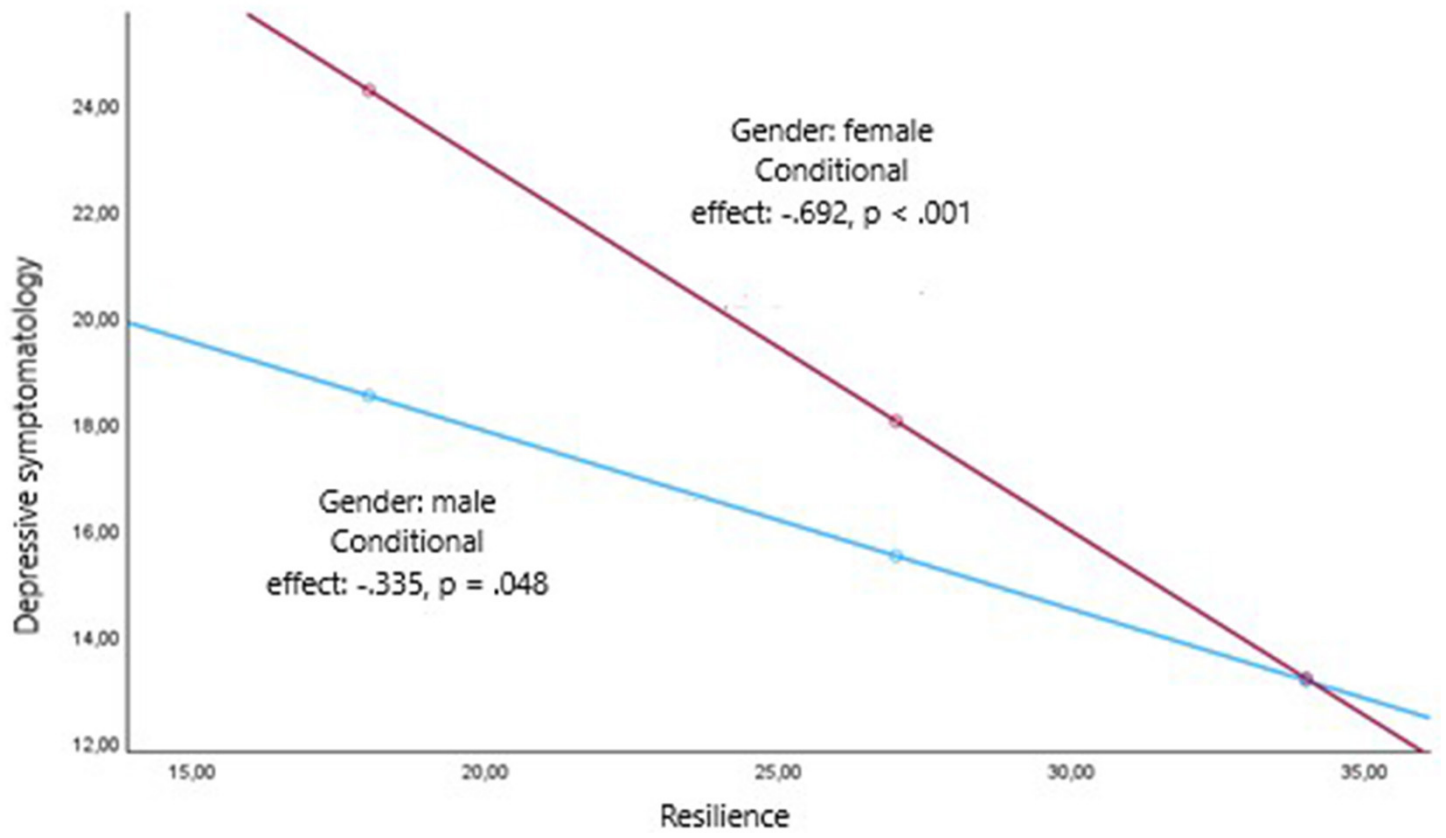
Conditional effects of resilience on the levels of the moderator variable gender of the caregiver.

**Figure 2:**
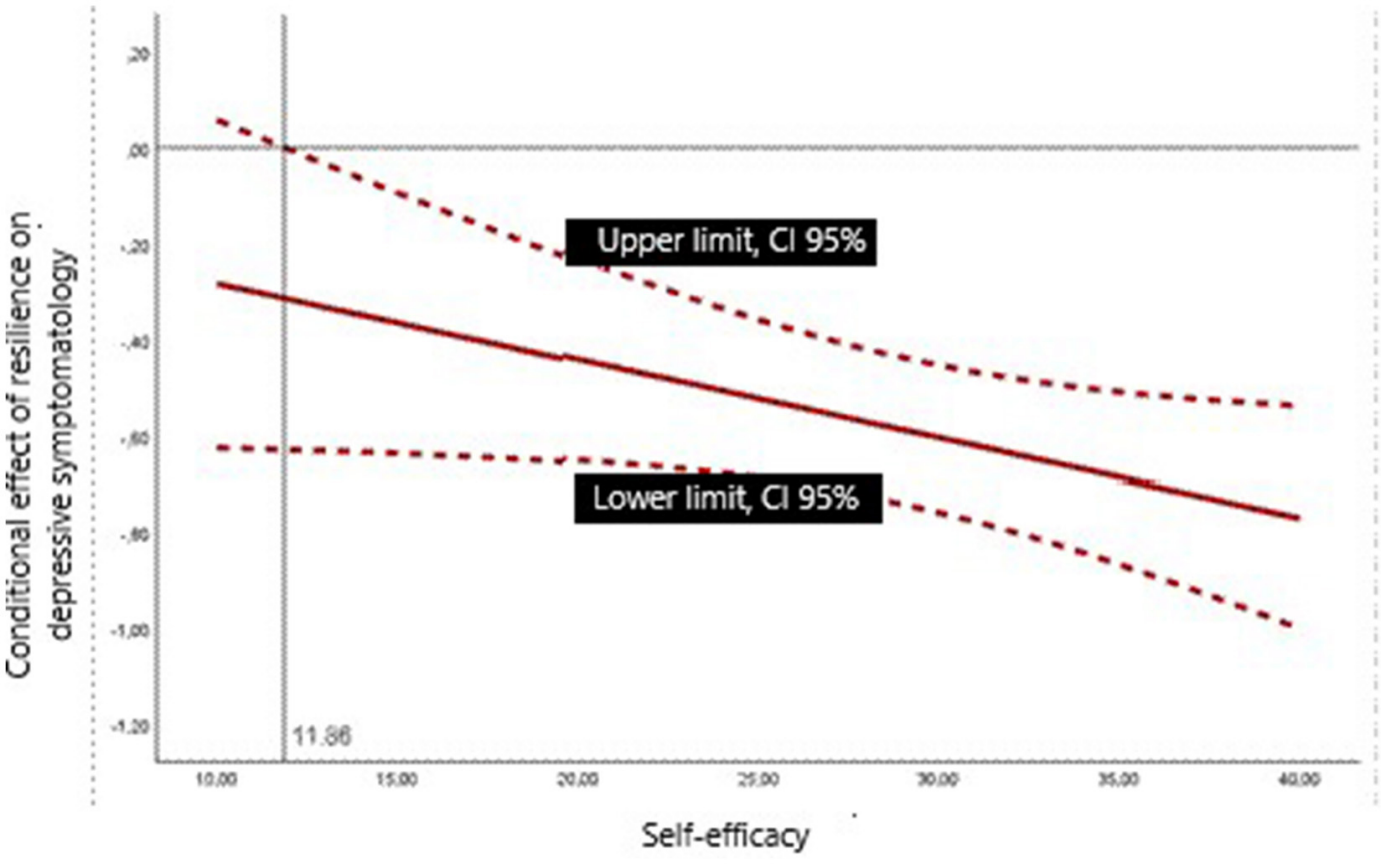
Conditional effect of resilience on depressive symptomatology based on the moderator variable self-efficacy.

Resilience was a predictor of the level of depressive symptomatology, such that a higher level of resilience was associated with lower depressive symptoms (*B* = −0.66, 95% CI [−0.77, −0.55], β = −0.44, *p* < 0.001). This regression model is presented in the first section of Table 6. Two moderating variables were found for the effect of resilience on depressive symptoms (see Table 6): gender of the informal caregiver and self-efficacy. The coefficient for the TM interaction between informal caregiver gender and resilience, *b*3 = −0.357, was significantly different from zero: *t*_(550)_ = −1.99, *p* = 0.047. Thus, being a man or woman can affect the relationship between resilience and depressive symptomatology. Figure 1 graphically represents this interaction. The slope associating resilience with depressive symptomatology was negative in both gender groups, indicating that higher informal caregiver resilience is associated with lower depressive symptoms in either category. However, it was more pronounced in female informal caregivers. The conditional effect estimates that an increase in one unit of resilience leads to a greater reduction in depressive symptoms when the informal caregiver is a woman. Similarly, self-efficacy moderated the relationship between resilience and depressive symptoms. The coefficient for the interaction, *b*3 = −0.016, was significantly different from zero: *t*_(550)_ = −2.01, *p* = 0.045. Figure 2 represents the regions delineated by the upper and lower limits of the effect, estimated using the Johnson-Neyman technique (Hayes, 2018, 2022). The effect extends to virtually the entire range of self-efficacy values, as it is estimated to occur from 11.86, a measure exceeded by 99.46% of the sample. For greater precision, we estimated the conditional effect of resilience on depressive symptomatology by simultaneously considering the two moderating variables, gender of the informal caregiver and self-efficacy in an additive multiple moderation model. Since it was plausible from a conceptual perspective, we also tested for a three-way interaction resilience ^*^ caregiver gender ^*^ self-efficacy, which was not statistically significant. Again, from Table 6 we can interpret coefficient *b*_4_ for gender of the informal caregiver and coefficient *b*_5_ for self-efficacy. In the additive multiple moderation model, coefficient *b*_4_ estimated the change in the effect of resilience on depressive symptomatology when the gender variable (male vs. female) was considered, holding the level of self-efficacy constant. Both coefficient *b*_4_ = −0.380, *t*_(548)_ = −2.124, *p* = 0.034, and coefficient *b*_5_ = −0.017, *t*_(548)_ = −2.037, *p* = 0.042 were statistically different from zero, which means that both variables moderated the effect of resilience on depressive symptomatology.

**Table 6:** Regression models of depressive symptomatology on resilience and moderating variables.

**Effects of the predictor variables**	**Coefficient**		***SE***	***t***	***p***	***CI 95%***	
						**Lower limit**	**Upper limit**
**Resilience**							
*R*^2^ = **0.196**							
*F*_(1, 552)_ = **134.403**, ***p***<**0.001**							
Intercept	*i_*Y*_*	35.688	1.566	22.790	< 0.001	32.612	38.764
Resilience (*T*)	*b*_1_	−0.663	0.057	−11.593	< 0.001	−0.775	−0.550
**Resilience** ^*^Gender of the informal caregiver							
*R*^2^ = **0.205**							
*F*_(3, 550)_ = **47.256**, ***p***<**0.001**							
Intercept	*i_*Y*_*	24.538	5.019	4.889	< 0.001	14.679	34.397
Resilience (*T*)	*b*_1_	−0.335	0.169	−1.985	0.048	−0.667	−0.003
Gender of the informal caregiver (*M*)	*b*_2_	12.182	5.284	2.306	0.022	1.803	22.560
Interaction *T* x *M* (*TM*)	*b*_3_	−0.357	0.180	−1.990	0.047	−0.710	−0.005
**Resilience** ^*^Self-efficacy							
*R*^2^ = **0.204**							
*F*_(3, 550)_ = **47.014**, ***p***<**0.001**							
Intercept	*i_*Y*_*	26.427	5.916	4.467	< 0.001	14.807	38.047
Resilience (*T*)	*b*_1_	−0.121	0.249	−0.486	0.627	−0.610	0.368
Self-efficacy (*M*)	*b*_2_	0.277	0.219	1.264	0.207	−0.154	0.708
Interaction *T* x *M* (*TM*)	*b*_3_	−0.016	0.008	−2.005	0.045	−0.032	−0.0003
**Resilience** ^*^Gender of the informal caregiver ^*^Self-efficacy							
*R*^2^ = **0.214**							
*F*_(5, 548)_ = **29.804**, ***p***<**0.001**							
Intercept	*i_*Y*_*	14.755	7.629	1.934	0.054	−0.229	29.740
Resilience (*T*)	*b*_1_	0.238	0.300	0.794	0.427	−0.351	0.828
Gender (*M*_1_)	*b*_2_	12.790	5.270	2.427	0.016	2.438	23.142
Self-Efficacy (*M*_2_)	*b*_3_	0.272	0.219	1.243	0.214	−0.158	0.701
Interaction *T* x *M*_1_ (*TM*_1_)	*b*_4_	−0.380	0.179	−2.124	0.034	−0.732	−0.029
Interaction *T* x *M*_2_ (*TM*_2_)	*b*_5_	−0.017	0.008	−2.037	0.042	−0.032	−0.001

SE, Standard error; CI, Confidence interval.

**Figure 3:**
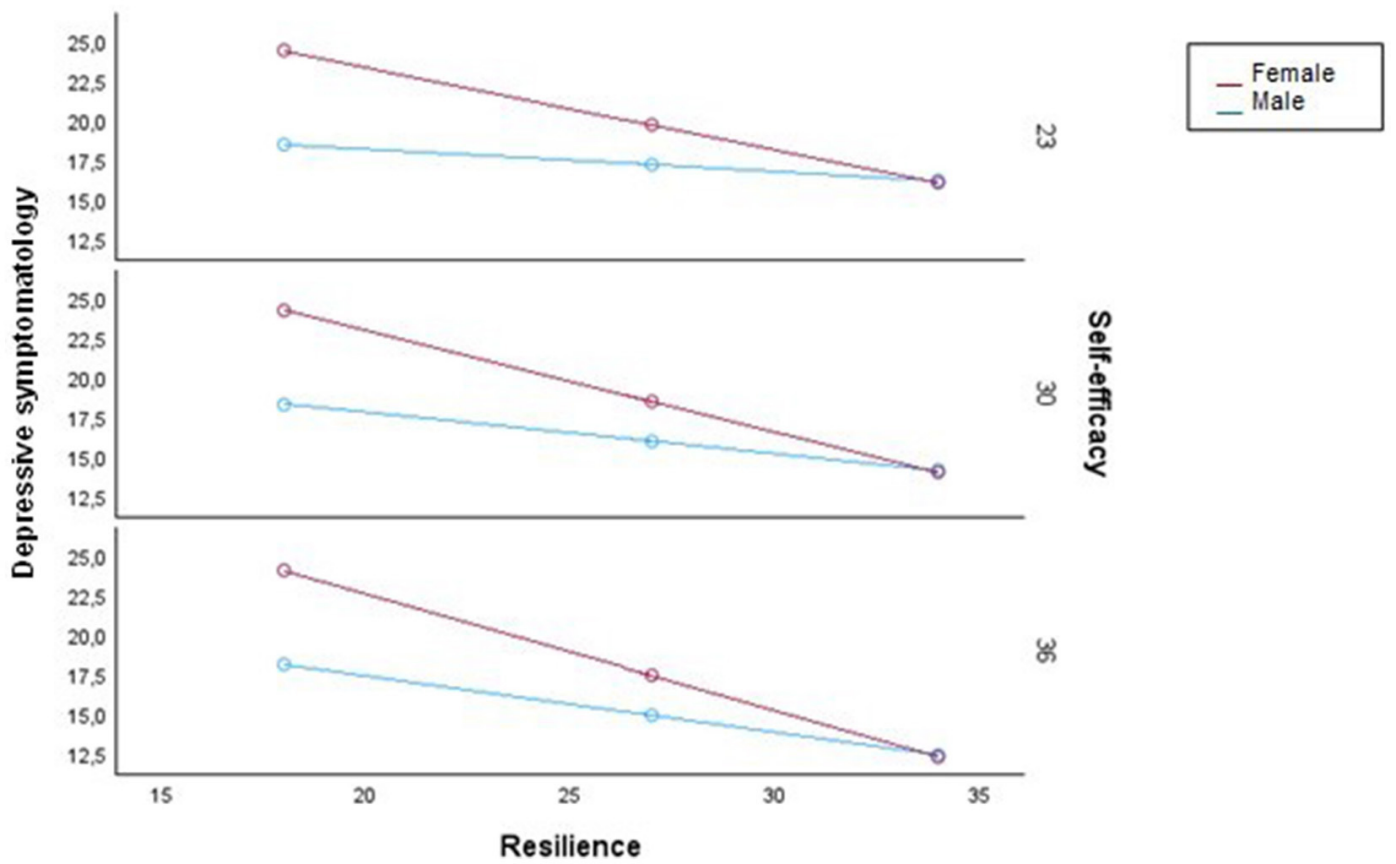
Conditional effect of resilience on depressive symptomatology as a function of gender of the caregiver and self-efficacy from the additive multiple moderation model.

Figure 3 is a visual representation of the additive multiple moderation model. The conditional effect of resilience on depressive symptomatology was different for men and women, and this distance held for different levels of self-efficacy. The three levels of self-efficacy shown in Figure 3 correspond to the 16^th^, 50^th^, and 84^th^ percentiles of the variable distribution.

The other sociodemographic variables of the informal caregiver (age, marital status, education level, main activity, and family monthly income), characteristics of the person in the situation of dependence (gender and age), characteristics of the caregiving situation (relationship of the informal caregiver to the person cared for, diagnosis of the care recipient, years of duration of the caregiving situation, and daily hours dedicated to caregiving), and clinical variables (positive environmental reward, negative thoughts, and personality variables [neuroticism, extraversion, and psychoticism]) did not moderate the relationship between resilience and depressive symptomatology.

## 4 Discussion

The objective of the current study was to determine the relationship between resilience and depression and its role as a protective factor against depression in informal caregivers, as well as the variables related to caregiving and clinical variables that could act as moderators in the relationship between resilience and depression.

The sample consisted of 554 informal caregivers of dependent individuals. The sociodemographic profile was predominantly female; in middle adulthood; married or living with a partner; with primary education; dedicated to domestic tasks and not engaged in paid work outside the home; and with a monthly family income between 1,000 and 1,999 €. This profile was consistent with previous international studies (e.g., Triantafillou et al., 2010; Colombo et al., 2011; Verbakel et al., 2017; OECD, 2021). However, some differences were noted. Notably, the percentage of women was higher than that found in several previous studies (Triantafillou et al., 2010; Colombo et al., 2011; Verbakel et al., 2017; OECD, 2021). This finding could be attributed to cultural differences. Similarly, most of the informal caregivers in the sample used in the present study had primary education, whereas in the study by Verbakel et al. (2017), the predominant educational level was secondary education. Lastly, although different average wages make it difficult to compare monthly income levels between countries, caregivers of working age are at a higher risk of poverty in all regions except Southern Europe (Colombo et al., 2011).

The profile of the care recipient was also that of a woman, with a mean age of 61.3 years; while the findings regarding the gender of the care recipient are consistent with the scientific literature, the care recipients in our study are younger than those in previous studies, where individuals over 70 or even 80 years old predominated (e.g., Otero et al., 2019; OECD, 2021). Regarding the relationship to the care recipient, about two-thirds of caregivers provide care to a parent or spouse, but there are age differences; younger individuals (between 50 and 65 years old) are more likely to care for a parent, while those over 65 are more likely to care for their spouse (OECD, 2021). Lastly, most informal caregivers cared for a care recipient with a dementia and had done so for almost 13 years, with a very high intensity. The importance of dementia as a cause for the need for care is consistent with previous research (e.g., Colombo et al., 2011; OECD, 2021). The duration of caregiving aligns with the range found in previous European scientific literature (Triantafillou et al., 2010; Otero et al., 2019; Vázquez et al., 2020). Regarding the daily hours dedicated to caregiving, there are significant differences between countries. Generally, around 50% of caregivers devote < 10 h per week, especially in Northern European countries and Switzerland, where < 20% provide care for more than 20 h a week. In contrast, in Southern European countries, the Czech Republic, and Poland, more than 30% of caregivers provide care for more than 20 h a week, and in Spain, this figure exceeds 50% (Colombo et al., 2011).

Regarding clinical variables, the mean score for positive environmental reward was similar to that found by Vázquez et al. (2019) in a sample of non-professional caregivers with similar characteristics and slightly lower than that found in the non-clinical Spanish population (Barraca and Pérez-Álvarez, 2010). These results are consistent with previous findings indicating that caregivers reduce their participation in pleasant and social activities (Labrum and Newhill, 2021). The mean score for negative thoughts was similar to that found by Otero et al. (2017) in a sample of informal caregivers of people with various health conditions. It was equivalent to that found in the original normative sample of university students (Hollon and Kendall, 1980), and similar or slightly lower than those found in other validation studies of the instrument in samples of workers (Deardorff et al., 1984) and clinical samples of depressed and non-depressed patients with mental or medical problems (Harrel and Ryon, 1983). The mean score for self-efficacy was similar to that found by Blanco et al. (2019b) in a sample of informal caregivers, and also within the range obtained for 25 countries by Scholz et al. (2002). Lastly, the mean scores for neuroticism, extraversion, and psychoticism were also similar to those found in previous studies with university students from various countries (Francis et al., 1992; Sandín et al., 2002).

The prevalence of depression was 16.1%. This figure is almost four times the weighted point prevalence of 4.7% globally found in the general population in a review and meta-analysis (Ferrari et al., 2013), which selected studies that used DSM or ICD criteria for the diagnosis of major depressive disorder. Although there are few previous studies that have used DSM-5 diagnostic criteria in the informal caregiver population, the prevalence in this study is higher than in previous research in populations of Spanish informal caregivers in which DSM-5 diagnostic criteria were used (10%; Torres et al., 2015, 2020) and similar to the prevalence in a sample of Hispanic informal caregivers of people with dementia in the United States (Cucciare et al., 2010). The most frequently reported symptoms in the present study for informal caregivers with major depression were depressed mood, insomnia, fatigue or loss of energy, and thoughts about death. In a previous study in the general adult population (Korten et al., 2012), it was also found that depressed mood and fatigue or loss of energy were very common symptoms, while insomnia appeared in between 41.7% and 52.2% of adults with depression and thoughts about death in between 62.1% and 65.4%; in both cases, these figures are much lower than those found in the current study. The predominance of depressed mood and insomnia as the most reported symptoms and the percentage of informal caregivers who report fatigue or loss of energy (i.e., higher than 70%) is consistent with previous studies (i.e., Torres et al., 2015, 2020). However, in the current study, 84.3% of the participations endorsed the symptom of having thoughts about death, which was higher than the 77.8% and 79.5% found in the studies by Torres et al. (2015, 2020). Likewise, more than half of the informal caregivers in the current study were at risk of depression. This figure is five times more than what was found by Pietrzak et al. (2013) in a sample of 34,654 adults (11.6% were at risk) from the general population in the United States; and much higher than the 41.4% found in previous research in informal caregivers (Vázquez et al., 2019).

The finding that one in six informal caregivers has clinical depression, and more than half are at risk of developing depression, reflects a significant increase in depression rates in recent years. A tentative hypothesis for this finding is that these data reflect the increase in depression figures in the general population derived from the COVID-19 pandemic found in previous studies (e.g., Wister et al., 2022). Additionally, the high rates of insomnia may be specific to the informal caregiver population and be due in part to the behavioral problems experienced by a large part of the people in a situation of dependency. In this regard, previous studies indicate that informal caregivers show more alterations in sleep quality, a reduction in total sleep time, and more fragmented sleep, compared to non-caregivers (Gao et al., 2019; Simón et al., 2022). Regardless, the percentage of caregivers who reported thoughts about death is much higher than previous research with samples of caregivers and those with a general population sample and requires special attention.

Although the criteria for determining the level of resilience in previous studies are diverse, which complicates the comparability of the findings, it should be noted that a significant percentage of informal caregiver participants in the current study reported a low level of resilience. Specifically, more than twice the proportion of participants reported low resilience compared to previous studies (e.g., Saria et al., 2017; Blanco et al., 2020). However, the low levels of resilience were similar to those reported in a study among informal caregivers of people with dementia, where about 75% had low resilience (Fernández-Lansac et al., 2012). The results from the current study highlight the difficulty of caregivers to positively adapt to their caregiving situation, especially during difficult periods of time. For example, the most frequently endorsed items of the CD-RISC-10 scale were: *Trying to recover after an illness or difficulty, Being able to adapt to changes*, and *Seeing myself as a strong person*. This positive perception of at least some of their own capacities may allow them to conduct their caregiving work more successfully, considering the many unforeseen events, changes, and sacrifices involved, often over prolonged periods.

Average resilience scores were higher for men, older informal caregivers, and those who had been caring for a longer period of time. This finding of higher resilience in men is consistent with some previous results (Joling et al., 2016), but are inconsistent with other previous research (Cherry et al., 2013; Dias et al., 2015; Teahan et al., 2018). A potential explanation is that the men in this sample might have chosen caregiving more voluntarily, unlike women, who might perceive caregiving as an obligation linked to their gender (Lee, 2010). There are also gender differences in the type and intensity of caregiving tasks, with women typically responsible for more demanding and regular tasks and investing more time daily and over more years (Jenson and Jacobzone, 2000; García-Calvente et al., 2004, 2011). Joling et al. (2016) also found a relationship between older age and higher resilience; however, other previous research has indicated that younger informal caregivers exhibit higher levels of resilience (Zauszniewski et al., 2010; Teahan et al., 2018). In this study, a possible explanation for older informal caregivers showing greater resilience is that they predominantly cared for their parents, a situation perceived as more normative, and thus potentially less stressful compared to caring for their children. Finally, caregiving duration has been associated with both higher (Dias et al., 2015) and lower (Teahan et al., 2018) resilience levels; the results of the current study support the hypothesis that the longer the caregiving duration, the higher the likelihood of successfully adapting to the informal caregiver role.

Furthermore, higher positive environmental reward, self-efficacy, and extraversion were associated with higher resilience; more negative automatic thoughts and neuroticism with lower resilience. These results are intuitive and coincide with previous research findings that found a direct relationship of positive psychological resources like self-efficacy (e.g., Fernández-Lansac et al., 2012; Dias et al., 2015) and positive personality traits like extraversion (e.g., Fernández-Lansac et al., 2012), and an inverse relationship with self-efficacy to control negative thoughts (Crespo and Fernández-Lansac, 2015) and negative personality traits like neuroticism (e.g., Fernández-Lansac et al., 2012; Dias et al., 2015). To our knowledge there are no studies that have examined environmental reward, but a tentative hypothesis is that greater reward received from the environment (e.g., in terms of recognition of the work they do) is related to better adaptation to stress, reinforcing the idea that the level of activity and social support contributes to strengthening psychological resources and is important for the mental health of the informal caregiver themselves (e.g., Atoyebi et al., 2022).

Overall, greater resilience predicted lower depressive symptoms, serving as a protective factor against depression. This finding is consistent with previous research (Dias et al., 2016; Pastor-Cerezuela et al., 2016; Sutter et al., 2016; Halstead et al., 2018; Bermejo-Toro et al., 2020). The moderating variables in the relationship between these two variables were gender and self-efficacy; it was found that, although there was a relationship between resilience and depression for all informal caregivers, resilience led to a greater reduction in depressive symptoms for female informal caregivers and the higher the self-efficacy, the greater the impact of resilience on depressive symptoms. Among informal caregivers with low levels of resilience, women experienced higher levels of depression than men and among informal caregivers with high levels of resilience, women experienced lower levels of depression than men. While there are no previous studies, to our knowledge, that have examined the moderators of resilience and depression in the informal caregiver population, a tentative hypothesis to this finding is that women tend to have lower resilience scores and higher depressive symptomatology than men overall. However, for those who adapt to the caregiving situation, caregiving is perceived as a source of purpose in life and makes them feel that they are fulfilling their obligations (Lee, 2010), resulting in greater emotional wellbeing. The finding regarding self-efficacy implies that the effect of resilience on depressive symptoms occurs at all levels of self-efficacy, except for a small percentage of informal caregivers who have no confidence in their ability to achieve their goals. Previous research has also identified a relationship between resilience and self-efficacy (e.g., Fernández-Lansac et al., 2012). The relationship between resilience and depressive symptoms differs between men and women at any level of self-efficacy. Similarly, the moderating effect of self-efficacy persists regardless of whether the caregiver is male or female.

Among the limitations of this study is the inability of the design to establish causal relationships between variables. Longitudinal studies with periodic assessments of the same individuals are required to explore how depressive symptomatology in informal caregivers changes over time. The use of an informal caregiver sample from Galicia might limit the generalizability of these findings. However, the profile of participants in this study resembles that observed in previous national and international research (e.g., Triantafillou et al., 2010; Colombo et al., 2011; Verbakel et al., 2017; OECD, 2021), suggesting the results may be broadly applicable.

The findings are significant for clinical practice and research, highlighting the need to develop protocols for detecting clinical symptoms and depression in this at-risk population and to create psychological intervention programs aimed at improving emotional wellbeing and preventing mental health issues. Specifically, psychological interventions should be developed to prevent depression in informal caregivers, promoting and enhancing a resilient coping style, focusing on identifying active coping strategies, and increasing awareness of the positive aspects of providing care, while simultaneously encouraging the search for personal meaning in the caregiving experience. Moreover, these programs should also promote self-efficacy to strengthen the impact of resilience on depressive symptoms, especially targeting female informal caregivers due to their higher vulnerability to depression. Lastly, it's crucial to urgently implement psychological and psychiatric interventions to prevent depression among the substantial number of informal caregivers currently suffering from it, as this seriously compromises their mental health and the care of their loved one.

## Data availability statement

The datasets presented in this article are not readily available because the study was conducted in accordance with the principles of the Declaration of Helsinki and ensured compliance with Organic Law 3/2018, of December 5, on Personal Data Protection and guarantee of digital rights. Requests to access the datasets should be directed to the data supporting the reported results can be requested from the Office for Gender Equality (OIX) at the University of Santiago de Compostela; oix@usc.es.

## Ethics statement

The studies involving humans were approved by the Bioethics Committee of the University of Santiago de Compostela. The studies were conducted in accordance with the local legislation and institutional requirements. The participants provided their written informed consent to participate in this study. Written informed consent was obtained from the individual(s) for the publication of any potentially identifiable images or data included in this article.

## Author contributions

FV: Conceptualization, Funding acquisition, Methodology, Project administration, Supervision, Writing – original draft, Writing – review & editing. VB: Conceptualization, Formal analysis, Investigation, Methodology, Writing – original draft, Writing – review & editing. EA: Conceptualization, Formal analysis, Methodology, Writing – review & editing. PO: Conceptualization, Investigation, Methodology, Writing – review & editing. AB: Conceptualization, Writing – review & editing. MS: Conceptualization, Writing – review & editing. AT: Conceptualization, Writing – review & editing.
